# Controlled-release fertilization reshapes maize yield through plant-soil synchrony: shifting from nitrogen supply intensity to synchronization-driven vegetative remobilization

**DOI:** 10.3389/fpls.2026.1901117

**Published:** 2026-08-26

**Authors:** Hongyu Ma, Xianyue Li, Mauro De Feudis, Qi Hu, Yanan Sun, Yue Li, Xinlu Li, Hongxing Liu, Gloria Falsone, Marcello Di Bonito

**Affiliations:** 1 State Key Laboratory of Water Engineering Ecology and Environment in Arid Area, Inner Mongolia Agricultural University, Huhhot, China; 2 Department of Agricultural and Food Sciences, Alma Mater Studiorum-Università di Bologna, Bologna, Italy; 3 Key Laboratory of Eco-Hydrology and Efficient Use of Water Resources of Inner Mongolia Autonomous Region, Hohhot, China; 4 College of Desert Control Science and Engineering, Inner Mongolia Agricultural University, Huhhot, China; 5 School of Animal, Rural and Environmental Sciences, Nottingham Trent University, Southwell, Nottinghamshire, United Kingdom

**Keywords:** controlled-release fertilizer, maize yield, nitrogen remobilization, nitrogen supply-demand synchronization, soil-plant nitrogen coupling, structural equation modeling

## Abstract

**Background and aims:**

Nitrogen (N) management in maize systems is constrained by low fertilizer use efficiency and substantial environmental losses, particularly in arid irrigated regions where soil N heterogeneity disrupts plant uptake and allocation. Controlled-release fertilizers (CRFs) offer a promising approach to coordinate soil N supply with crop N demand and regulate internal N cycling during yield.

**Methods:**

To elucidate the mechanisms by which CRF enhances N utilization and coordinates N allocation, field experiments were conducted in western Inner Mongolia, China from 2022 to 2024. This study to evaluate their impact on N accumulation (NA), tissue-specific N partitioning and N remobilization, leaf N remobilization efficiency (NRE_leaf_), stem N remobilization efficiency (NRE_stem_), supply-demand synchrony index (SDI), supply–plant N requirement synchronization (SPNR), soil nitrate dynamics, and maize yield throughout the growing season.

**Results:**

The results showed CRF markedly enhanced N remobilization, with NRE_leaf_ and NRE_stem_ increasing by 12.3% and 24.8%. Notably, CRFs also improved overall N supply-demand synchronization: average SDI values for CF3 and CF2 were 0.77 and 0.86, compared with 0.61 for regular fertilizer (RF), representing a 26-41% increase. Building on this, structural equation modeling revealed that the mechanism regulating yield shifted from N supply intensity under RF (R^2^= 0.74) to synchronization-driven control under CRF (R^2^ = 0.79), in which SPNR coordinated N allocation and remobilization (>0.8 contribution) to determine grain NA and final yield.

**Conclusion:**

These findings demonstrate that optimizing N supply-demand synchronization, rather than increasing N inputs, fundamentally reshapes maize yield formation by coordinating internal N cycling. CRFs, particularly at moderate N rates, offer a pathway for improving N use efficiency while reducing environmental risk, providing actionable insights for sustainable maize production in N-limited, arid irrigated systems.

## Graphical Abstract

**Figure:**
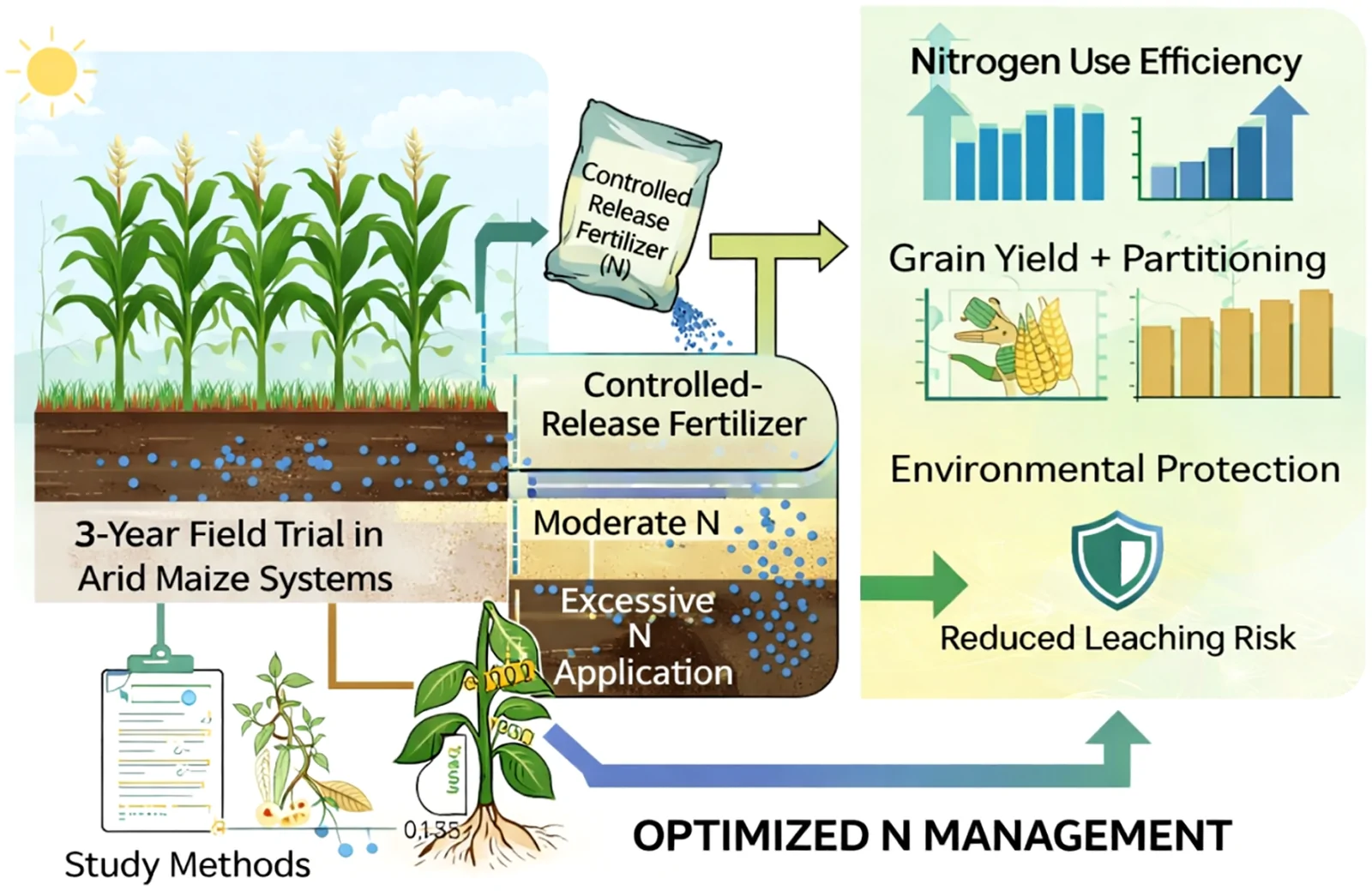

## 1 Introduction

Nitrogen (N) is a key determinant of maize productivity because it is essential for the synthesis of proteins, nucleic acids, and chlorophyll, thereby regulating dry matter accumulation and seed yield (Asibi et al., 2019; Wani et al., 2021). The global expansion of N fertilizer application has significantly boosted agricultural productivity; however, its widespread use is often inefficient, with less than 50% of applied N being effectively taken up by crops (Martínez-Dalmau et al., 2021). The remaining N is frequently lost to the environment through leaching, volatilization, and denitrification, contributing to groundwater contamination, greenhouse gas emissions, and eutrophication of aquatic systems (Bijay-Singh and Craswell, 2021; Byrnes, 1990). Understanding how maize physiologically responds to N distribution, especially in terms of N uptake and partitioning among plant organs, is essential for improving N use efficiency (NUE) and designing spatially optimized fertilization strategies (Duan et al., 2023).

Controlled-release fertilizers (CRFs) particularly polyurethane-coated urea and other polymer-coated formulations, have been increasingly applied in maize systems to synchronize N supply with plant demand (Jariwala et al., 2022) compared to conventional urea CRFs have consistently been shown to reduce N leaching, ammonia volatilization, and nitrous oxide emissions while maintaining or enhancing maize yield performance (Gao et al., 2021; Li et al., 2021; Sun et al., 2024). Field trials have also demonstrated that CRFs optimize the temporal and spatial availability of mineral N in the root zone and enhance N uptake during critical growth stages (Qiang et al., 2020; Zhang et al., 2023b). N mass balance approaches have evaluated N input, uptake, residuals, and loss pathways under CRF management, offering insights into soil–plant N cycling efficiency (Ji et al., 2021; Li et al., 2020; Zhou et al., 2019).

Despite these knowledge advances, little is known about how different CRFs formulations influence N allocation at the organ and tissue levels in maize (Jariwala et al., 2022; Lee and Ku, 2025). Most existing studies quantify total plant N uptake, recovery efficiency, often neglecting the redistribution of N among soil, stems, leaves, and grains, especially under heterogenous soil N conditions (Li et al., 2013). In terms of N distribution within the plant, CRFs have shown potential to influence partitioning of N to specific organs such as leaves, stalks, and grains across developmental stages, although such effects remain underexplored compared to yield and NUE endpoints (Ren et al., 2025a; Liu et al., 2024; Wicaksono et al., 2022).

In maize production systems, crop growth and yield are strongly influenced by the coordination between soil N availability, plant N uptake, tissue-level N redistribution, and the synchronization of soil N supply with crop N demand, all of which collectively regulate partial factor productivity of N (PFP), N agronomic efficiency (NAE), grain filling, and final yield formation under different fertilization strategies (Liu et al., 2022). The physiological basis of improved PFP and NAE with CRFs—such as enhanced N translocation, remobilization during grain filling, or preferential partitioning to reproductive tissues—remains poorly understood (Ji et al., 2021; Qiang et al., 2020). In particular, it remains few studied the relationships between controlled N release, root-zone nitrate availability, tissue-level N redistribution, and grain N accumulation (Li et al., 2020). A better understanding of these processes is needed to explain how CRFs improve PFP, NAE, and yield formation beyond simply increasing N availability.

To address this knowledge gap, a 3-years field experiments were conducted in a typical arid irrigated maize production area in western Inner Mongolia, China, comparing conventional urea with different CRF application rates. The study integrated seasonal monitoring of soil N dynamics with tissue level N allocation and remobilization analyses to investigate the mechanisms linking controlled N release, soil-plant synchronization, and crop productivity. The findings will contribute to optimizing N management and enhancing N use efficiency in water-limited cropping systems. The objectives of this study were as follows: (1) quantify the effects of CRF versus conventional urea on maize N use, including partial factor productivity (PFP), N agronomic efficiency (NAE), tissue-specific N accumulation, and remobilization dynamics; (2) assess how N management and fertilizer type influence soil nitrate distribution and its interaction with plant N uptake; (3) evaluate the synchronization between soil N supply and plant N demand under these fertilization strategies; (4) elucidate yield mechanisms under different fertilizers, showing how CRF shifts control from N supply intensity to synchronization-driven tissue N allocation and remobilization.

## 2 Materials and methods

### 2.1 Study area

**Figure 1:**
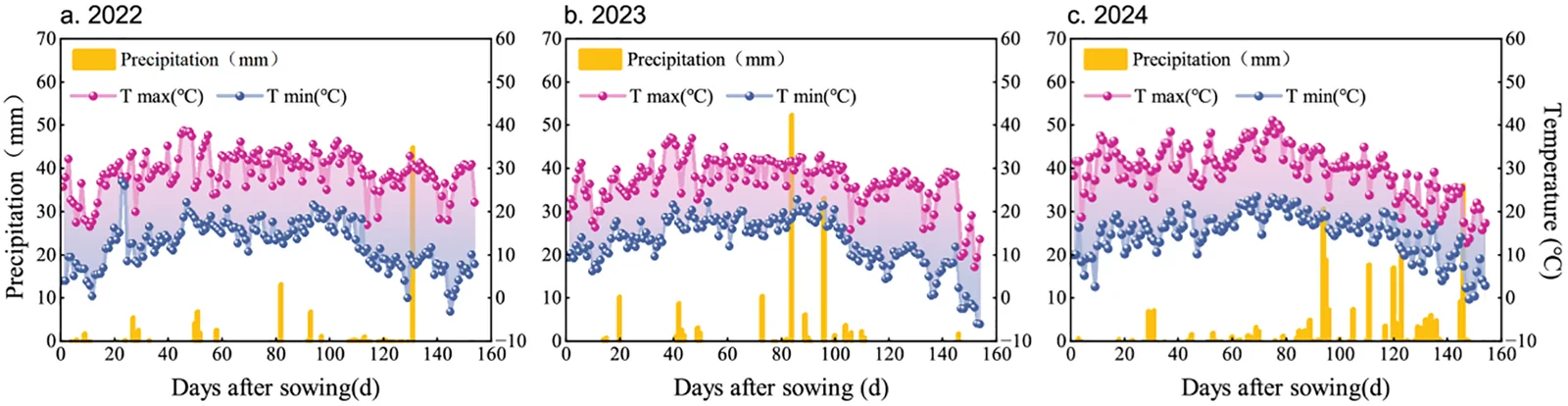
Temperature and precipitation during the spring maize growing seasons of 2022-2024.

This experiment was conducted from May 2022 to October 2024 in Lianxing Village, Wuyuan County, Bayannur City; the study area is located in Bayannur City, Wuyuan County, Lianxing Village, 8100 ha of saline and alkaline land improvement demonstration area (108.28° E, 41. 15° N; **Figure 1**), with a mesothermal semi-arid continental monsoon climate, light energy-rich, sunshine, dry and windy. The average annual temperature is 6.3-7.7 °C; the annual sunshine time is 3263 ah; and the frost-free period is 117-136d, which is relatively short. The average total annual solar radiation is 153.44 cal cm^-2^, and the cumulative temperature is 3,362.5 °C; the average annual potential evaporation is about 1900 mm; the average annual rainfall is 170 mm, mostly concentrated in summer and autumn, with rain and heat in the same season. The main soil type of the western region is classified as powder loam soil. The soil organic matter, total N, quick-release potassium, quick-release N, electrical conductivity (EC), soil pH and bulk density of cultivated soil layer (0–20 cm) were 5.102%, 0.90 ag kg^-1^, 163 mg kg^-1^, 78 mg kg^-1^, 1.34 dS m^-1^, 8.45 and 1.46 ag cm^-3^. A general weather station was set up to record climatic variables during the two growing seasons of maize (**Figure 1**).

### 2.2 Experimental design

During the test, the crop chosen was maize, variety JunKai MK360, planted by mechanical seeding, with a pattern “one film, one tube, two rows”, with a film width of 80 cm, a row spacing of 40 cm, and a planting density of 66,670 plants ha^-1^. The seeds were sown on 2 May 2022, 10 May 2023 and 8 May 2024. They were harvested on 1 October 2022, 3 October 2023 and 6 October 2024, in that order. The experiment had four different N application treatments, including N regular fertilizer (225 kg ha^-1^, RF), and controlled-release low N (150 kg ha^-1^, CF1), medium N (225 kg ha^-1^, CF2), and controlled-release high N (300 kg ha^-1^, CF3). In addition, a control treatment with no N fertilizer (0, NF) was included (**Table 1**). The field experiment was conducted using a randomized complete block design (RCBD) with three blocks. Each block contained five N treatments, which were arranged within each block according to a randomized complete block design. Each treatment was replicated three times. The experimental plot (18 m×4 m) was considered as the experimental unit for statistical analysis. The CRF treatments were applied to the field by a single basal application of polymer-coated urea (28% N), manually applied after sowing. The regular fertilizer was diammonium phosphate (14% N) as a basal fertilizer applied to the field at the time of mulching; the follow-up fertilizer was urea (46% N) applied manually before irrigation at the end of the nodulation period, and the fertilizer was applied at a position of 5 ± 0.5cm. All plots were managed using under-membrane drip irrigation during the maize growing season. Irrigation was applied according to crop water requirements and soil moisture conditions, with 7–10 irrigation events during the reproductive period. Each irrigation event supplied 20 mm water. The irrigation schedule was consistent among N treatments within each year.

**Table 1:** Irrigation and fertilizer treatments, planting dates, harvest dates and sampling times for maize farmland, 2022-2024.

Year	Date of transplanting	Date end of the maize	Duration (days)	Sampling period (DAT)^a^	Irrigation amount (mm)^b^	N rates (kg ha^-1^)	
						Controlled release fertilizer	Conventional fertilizer
2022	02-May	01-Oct	153	V6, V12, VT, R2 and R6	20	0-150-225-300	225
2023	10-May	03-Oct	146	V6, V12, VT, R2 and R6	20	0-150-225-300	225
2024	08-May	06-Oct	152	V6, V12, VT, R2 and R6	20	0-150-225-300	225

^a^DAT: V6=Sixth leaf stage, V12=Twelfth leaf stage, VT=Tasseling stage, R^2^ = Blister stage, R^6^ = Physiological maturity stage.

^b^The entire maize reproductive cycle.

### 2.3 Sampling and measurements

#### 2.3.1 Dry matter determination

At the VT, R2, and R6 stages, six representative plants were randomly selected from each experimental plot for biomass and N accumulation measurements. The values obtained from the six plants within each plot were averaged before statistical analysis. The experimental plot was considered as the experimental unit, and the three plots of each treatment were used as independent replicates. Plants were cut at the base of the stem, and the underground portions were discarded. Dust and debris were removed from the plant surface, after which stems, leaves, fruits, and kernels were separated and individually bagged. The samples were first oven-heated at 105 °C for 1 ah to halt metabolic activity, then dried at 75 °C to a constant weight. After cooling in a desiccator, samples were weighed using an electronic balance. Dry matter accumulation was calculated on a per-hectare basis and averaged across replicates (Li et al., 2024; Shu et al., 2022).

#### 2.3.2 Plant biomass and N determination

Maize plants were sampled at the VT, R2 and R6 stages. Six representative plants were cut at the base in each experimental plot. The plants were separated into leaf, stem, and grain components. Each component was oven-dried (105 °C for 1 ah, then 75 °C until a constant weight was reached) and weighed in order to calculate the dry matter content. Samples of ground leaves, stems and grains (0.5 mm) were analyzed for total N using the Kjeldahl digestion method (Bremner and Mulvancy, 1982). The N accumulation (NA) amounts for different plant parts were calculated as follows:

(1) Plant N accumulation was calculated by (Equation 1):

(1) $$NA({\text{kg ha}}^{-1})=B_{i}\times \text{plant N content}(\%)$$

Where *B_i_* is the plant dry biomass (kg ha^-1^).

The NA amounts for different plant parts were calculated as follows (Equation 2):

(2) $$NA\left(\text{grain}/\text{stem}/\text{leaf}\right)\left({\text{kg ha}}^{-1}\right)=Wi\left(\text{grain}/\text{stem}/\text{leaf}\right)\times N\left(\text{grain}/\text{stem}/\text{leaf}\right)\text{content}\left(\%\right)$$

(2) N partitioning indices (*NPI*):

To quantify the internal distribution of N among maize tissues, *NPI* were calculated at physiological in VT, R2, and R6 stage (Equation 3):

(3) $${NPI}_{i}=\frac{N_{i}}{\sum N_{total}}\times 100\%$$

Where *N_i_* is the N content of tissue (grain/stem/leaf) *i*, and $\sum N_{total}$ is the sum of N content in all aboveground tissues at harvest. This index reflects the relative allocation of N to different maize tissues, providing insights into N distribution efficiency under different treatments (Nehe et al., 2020).

(3) N remobilization and N remobilization efficiency (NRE) (Equations 4, 5):
  (4) $$N_{\text{rem, leaf}}=N_{\text{leaf, VT}}-N_{\text{leaf, R}6}$$
  (5) $$N_{\text{rem, stem}}=N_{\text{stem, VT}}-N_{\text{stem, R}6}$$

Where *N_rem,leaf_* and *N_rem,stem_* are N remobilization from leaves and stems, respectively (kg ha^−1^); *N_leaf,VT_* and *N_stem,VT_* are N accumulation in leaves and stems at the VT stage, respectively (kg ha^−1^); *N_leaf,R6_* and N*_stem,R6_* are N accumulation in leaves and stems at the R6 stage, respectively (kg ha^−1^). NA in plant tissues is calculated as the product of tissue dry matter and N concentration.

Total N remobilization (*N_rem,total_*) from vegetative tissues was calculated as follows (Equation 6):

(6) $$N_{\text{rem, total}}=\left(N_{\text{leaf, VT}}+N_{\text{stem, VT}}\right)-\left(N_{\text{leaf, R}6}+N_{\text{stem, R}6}\right)$$

Where *N_rem, total_* is the total amount of N remobilized from vegetative tissues (leaves and stems) to the grain during reproductive period (VT - R6).

N remobilization from vegetative tissues was quantified as the decrease in NA between the VT and R6 stages. NRE was calculated separately for leaves and stems to reflect tissue-specific N remobilization efficiency (NRE_leaf_ and NRE_stem_, %) according to Equations 7 and 8:

(7) $${\text{NRE}}_{\text{leaf}}=\frac{N_{\text{leaf, VT}}-N_{\text{leaf, R}6}}{N_{\text{leaf, VT}}}\times 100\%$$

(8) $${\text{NRE}}_{\text{stem}}=\frac{N_{\text{stem, VT}}-N_{\text{stem, R}6}}{N_{\text{stem, VT}}}\times 100\%$$

#### 2.3.3 Maize yield determination

During the maize maturation stage, 1m^2^ of maize was randomly selected from each plot for harvesting, threshing and cleaning. The cleaned maize grains were dried to a moisture content of 14%, which is the standard for safe storage. The dried grains were then weighed, and the yield per unit area was calculated based on the sample area and weight.

#### 2.3.4 Soil indicators determination

Soil samples were collected at 7–10 day intervals throughout the maize growing season to monitor dynamic changes in soil properties. Soil NO_3_^−^-N concentrations were determined at five key maize growth stages (V6, V12, VT, R2, and R6) for statistical comparisons and subsequent analyses. In addition, soil bulk density was measured annually before maize sowing at the corresponding soil depths and was used for calculating soil nitrate-N stocks.

##### 2.3.4.1 Soil bulk density

Soil bulk density (BD) was measured annually before maize sowing using the undisturbed soil core method. Separate undisturbed soil cores were collected from five soil layers (0-20, 20-40, 40-60, 60–80, and 80–100 cm), corresponding to the soil NO_3_^−^-N sampling depths. The soil cores were oven-dried at 105 °C until a constant weight was reached, and BD was calculated as the ratio of dry soil mass to soil core volume. The bulk density values measured for each soil layer and year were used for the calculation of soil nitrate-N stocks.

##### 2.3.4.2 Soil NO_3_^−^-N concentration

Each soil sample was divided into two parts. One part was used to measure soil moisture content using the oven-drying method. The other part was air-dried, crushed, mixed thoroughly, and passed through a 1 mm sieve. A 5 ag subsample was extracted with 50 mL of 2 M KCL solution by shaking for 1 ah on a reciprocating shaker. The extract was filtered through Whatman No. 42 filter paper, and the NO_3_^−^-N concentration was determined using a UV spectrophotometer (Cawse, 1967; Dong et al., 2014).

### 2.4 Calculation of N utilization efficiency index and N partitioning

Two N use efficiency indices were calculated to evaluate N uptake and utilization by maize: partial factor productivity of N (PFP, kg grain kg^-1^ N) and N agronomic efficiency (NAE, kg grain kg^-1^ N) (**Equations 9**, **10**).

(9) $$PFP=\frac{Y_{N}}{F_{N}}$$

(10) $$\text{NAE}=\frac{Y_{N}-Y_{0}}{F_{N}}$$

Where *Y_N_* is the maize yield following N fertilization (kg ha^-1^); *F_N_* is the amount of applied N; *Y_0_* is the crop yield (kg ha^-1^) under the no N application treatment.

### 2.5 Soil-plant N ratio calculation

To quantify the synchronization between soil N supply and crop N demand, a supply–demand synchrony index (SDI) was developed at key maize growth stages (V6, VT, R2, and R6). The concept of SDI was derived from the ecological principle that improving NUE requires synchronizing soil N availability with crop N demand during crop development (Crews and Peoples, 2005). Previous studies have demonstrated that temporal mismatches between soil N supply and crop N uptake are major causes of N losses and low N recovery efficiency. Therefore, evaluating the balance between soil N availability and plant N demand provides an important approach for understanding N management performance.

At each growth stage (V6, VT, R2, and R6), soil N supply (*S_t_*, kg ha^-1^) was represented by the cumulative NO_3_^−^-N content within the 0–100 cm soil profile, calculated as follows (Equation 11):

(11) $$S_{t}=\sum_{i=1}^{n}\left(C_{i}\times {BD}_{i}\times D_{i}\times 10\right)$$

where *C_i_* is the NO_3_^−^-N concentration (mg kg^-1^), *BD_i_* represent the measured soil bulk density of each experimental year (g cm^-3^), and *D_i_* is soil layer thickness (cm).

At the same growth stages, plant N demand (*D_t,_* kg ha^-1^) was represented by plant N accumulation (Equation 12):

(12) $$D_{t}=\sum_{j=1}^{m}\left(W_{j}\times N_{j}\right)$$

where *W_j_* is the dry biomass (kg ha^-1^), and *N_j_* is the N concentration (g kg^-1^) of each plant tissue (leaf, stem, and grain).

The SDI was defined as follows (Equation 13):

(13) $$SDI=1-\frac{1}{n}\sum_{t=1}^{n}\left|\frac{S_{t}-D_{t}}{max(D_{t})}\right|$$

The SDI provides an integrative indicator of soil–plant N coupling by directly comparing soil N availability with plant N uptake at the same growth stage. Higher SDI values indicate N surplus, whereas lower values indicate improved synchronization or potential N limitation.

Compared with SDI, which integrates the relative contribution of soil N availability and plant N accumulation across growth stages, supply–plant N requirement synchronization (SPNR) directly reflects the instantaneous balance between available soil N supply and crop N requirement at a specific developmental stage. Thus, SDI describes the dynamic synchronization trajectory, whereas SPNR provides a stage-specific assessment of N balance. The SPNR at each growth stage was calculated as follows (Equation 14):

(14) $$SPNR=\log\left(\frac{S_{t}}{D_{t}}\right)$$

The SPNR provides a direct indicator of soil–plant N balance at a given growth stage. SPNR>1: soil N supply exceeds plant N demand (potential N surplus); SPNR≈1: optimal synchronization between soil N supply and plant uptake; SPNR<1: plant N demand exceeds soil supply (potential N limitation).

### 2.6 Statistical analysis

Data calculations and statistical analyses were performed using RStudio (version 2024.3; RStudio Inc., Boston, MA, USA) and IBM SPSS Statistics (version 26.0; IBM Corp., Armonk, NY, USA). Origin (version 20.0; Origin Lab Corp., Northampton, MA, USA) was used for data analysis and visualization.

Statistical analyses were performed separately for each experimental year (2022-2024). Before conducting analysis of variance (ANOVA), data normality and homogeneity of variance were tested to confirm the suitability of parametric analysis. One-way ANOVA was conducted using SPSS to evaluate the effects of N treatments within each year. When significant differences were detected, mean comparisons were performed using the least significant difference (LSD) test at a significance level of 0.05.

Structural equation models (SEMs) were conducted in AMOS 21.0 (Ver. 21.0, SPSS Inc., Chicago, IL, USA) to evaluate the variables’ influence on maize yield, tissues N and their relationships. SEM interpretation included regression analysis, factor analysis, correlation analysis, and variance analysis. The final optimal model, selected for statistical significance, provides pathways for illustrating these interactions. The specific process is as follows:

1. Variable selection: The variables significantly related to yield were screened based on Pearson correlation coefficients (p< 0.05) (SDI, SPNR, NRE, NPI, leaf N content, stem N content, and yield). Fixed factors included in the model were further determined by referring to existing studies on corn N utilization.
2. Model setting: Maximum likelihood estimation (MLE) was applied, and the complete model was constructed using the AMOS 21.0 software for SEM. SDI and SPNR were included as composite indicators representing soil–plant N synchronization.
3. The goodness-of-fit of the SEM was assessed using χ^2^/df, goodness-of-fit index (GFI), and root mean square error of approximation (RMSEA). A satisfactory model fit was considered when χ^2^ test p > 0.05, RMSEA< 0.05, and GFI > 0.90. The significance of path coefficients was evaluated using 1000 bootstrap resampling procedures (p< 0.05). Residual analysis was performed to further examine model adequacy and error distribution.

## 3 Results

### 3.1 Effect of different CRF treatments on NAE, and PFP

**Figure 2:**
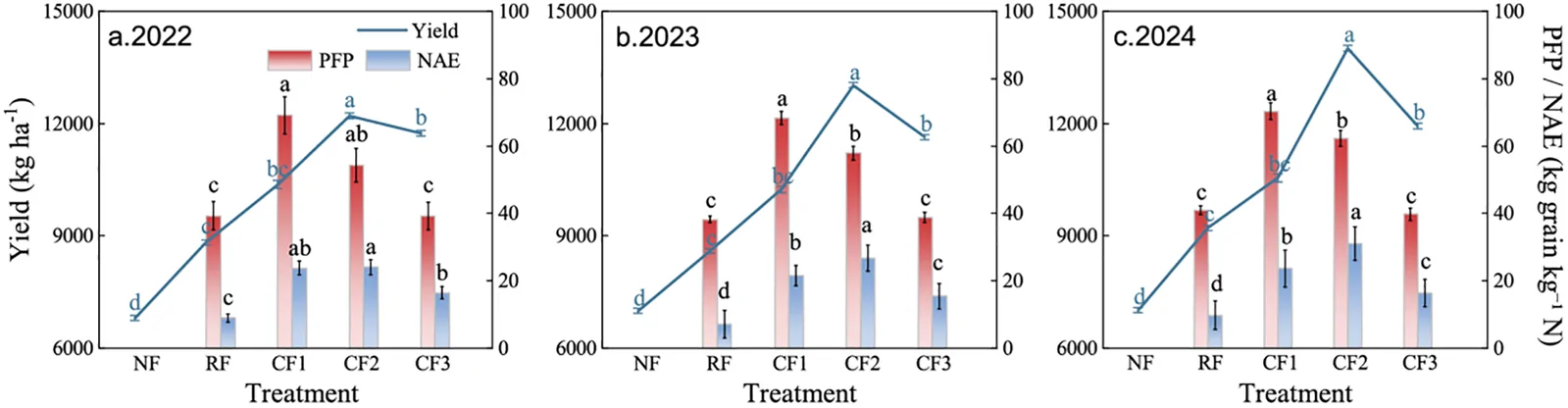
Effects of N treatments on maize yield, partial factor productivity of N (PFP), and N agronomic efficiency (NAE) in 2022 **(a)**, 2023 **(b)**, and 2024 **(c)**. Within each experimental year, different letters above the bars indicate significant differences among treatments at p< 0.05 according to the LSD test. NF = no N fertilizer; RF = 225kg ha^-1^ N of regular fertilizer; CF1 = 150kg ha^-1^ N of controlled-release fertilizer; CF2 = 225kg ha^-1^ N of controlled-release fertilizer; CF3 = 300kg ha^-1^ N of controlled-release fertilizer.

On the basis of the three-year average from 2022-2024 (**Figure 2**), the CF1 treatment was significantly better than the other fertilization treatments in terms of NUE. In terms of NAE, CF1 was 21%, 7% and 12% higher than RF, CF2 and CF3 treatments, respectively. Moreover, the PFP of CF1 treatment was 25.8%, 20.3% and 12.2% higher than that of RF, CF2 and CF3 treatments, respectively. CF1 showed higher NUE, NAE, and PFP compared with other fertilization treatments.

In 2023, all treatments achieved relatively high yield and NAE due to moderate rainfall. However, over-fertilized treatments, such as CF3, experienced a decrease in NAE by 18 kg grain kg^-1^ N in 2024 despite abundant rainfall, show that the risk of N loss increased under high rainfall conditions, which reduced the effectiveness of the CRFs.

### 3.2 N partitioning in maize tissues

**Figure 3:**
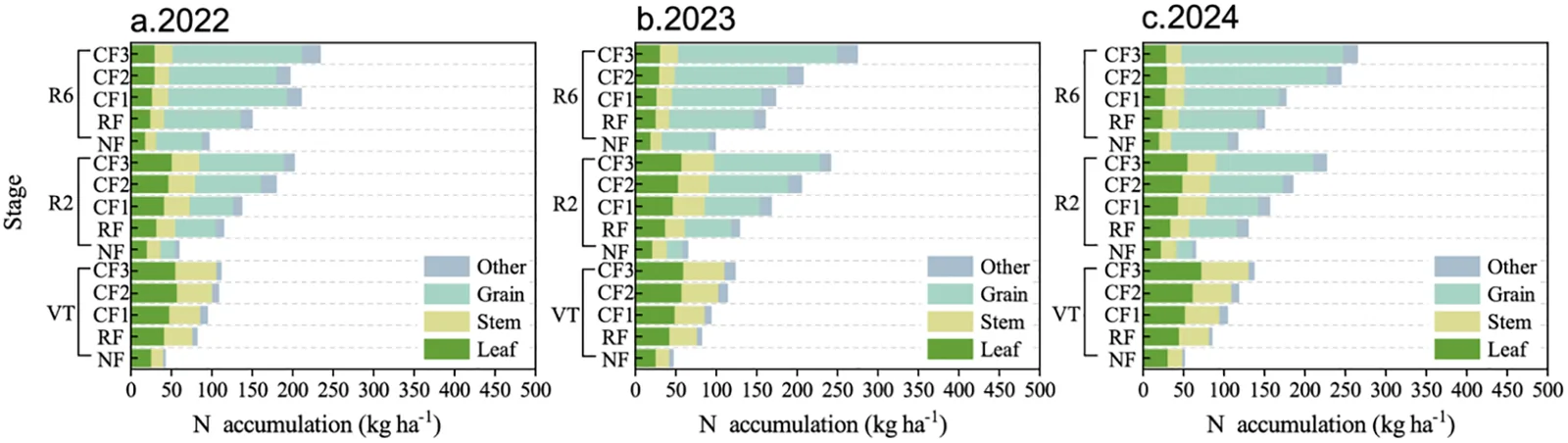
Tissue N distribution in 2022 **(a)**, 2023 **(b)** and 2024 **(c)**. NF = no N fertilizer; RF = 225kg ha^-1^ N of regular fertilizer; CF1 = 150kg ha^-1^ N of controlled-release fertilizer; CF2 = 225kg ha^-1^ N of controlled-release fertilizer; CF3 = 300kg ha^-1^ N of controlled-release fertilizer; VT=Tasseling stage, R2=Blister stage, R6=Physiological maturity stage.

At the R6 stage (**Figure 3**), the total N accumulation of CF treatment was on average about 45%-52% higher than that at the R2 stage, while the increase of NF treatment was only 30%. CRFs were better able to maintain N supply at the R2 stage to meet the grain filling demand.

Comparing different fertilizers at the same N application level, the CF2 treatment increased N retransfer from leaves and stems to grains by 15.3%, and N accumulation in grains by 20.0%, indicating that CRFs significantly enhanced NRE, which contributed to grain filling and yield formation.

**Figure 4:**
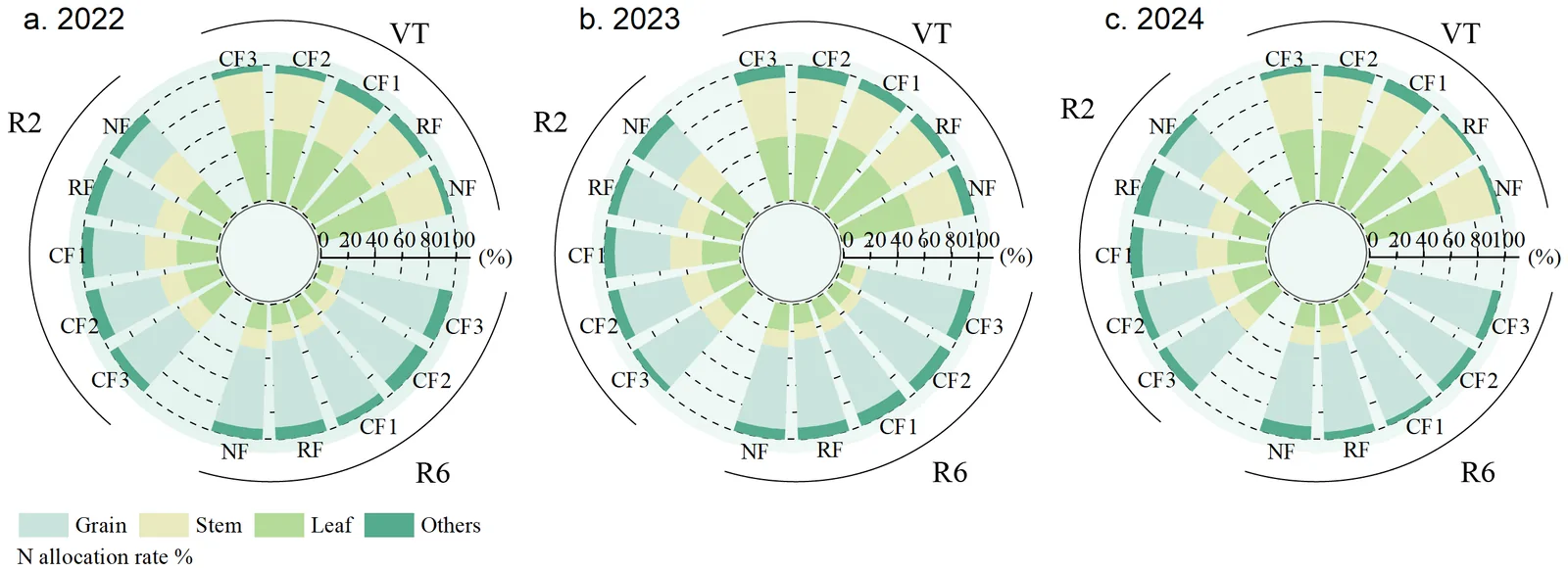
Proportion of N allocation to each tissue in 2022 **(a)**, 2023 **(b)** and 2024 **(c)**.

From the VT to the R6 stage (**Figure 4**), N allocation was characterized by a transfer from nutrient tissues to reproductive tissues, which was manifested by a continuous decrease of N in leaves and stems and a rapid accumulation of N in seeds. During the development from VT to R6 stage, the rate of seed N allocation increased significantly. In the CF2 treatment, for example, the proportion of grain N increased from 45.7% in the R6 stage to 67.3% in the R6 stage in 2022, an increase of 21.6%; in comparison, the RF treatment increased by 20% during the same period, which indicated that the CRFs significantly enhanced the N redistributing ability during the filling stage of the grains. At the R6 stage, CF2 and CF3 treatments were generally higher than RF and NF by 8%-12%, showing higher N reutilization efficiency.

**Figure 5:**
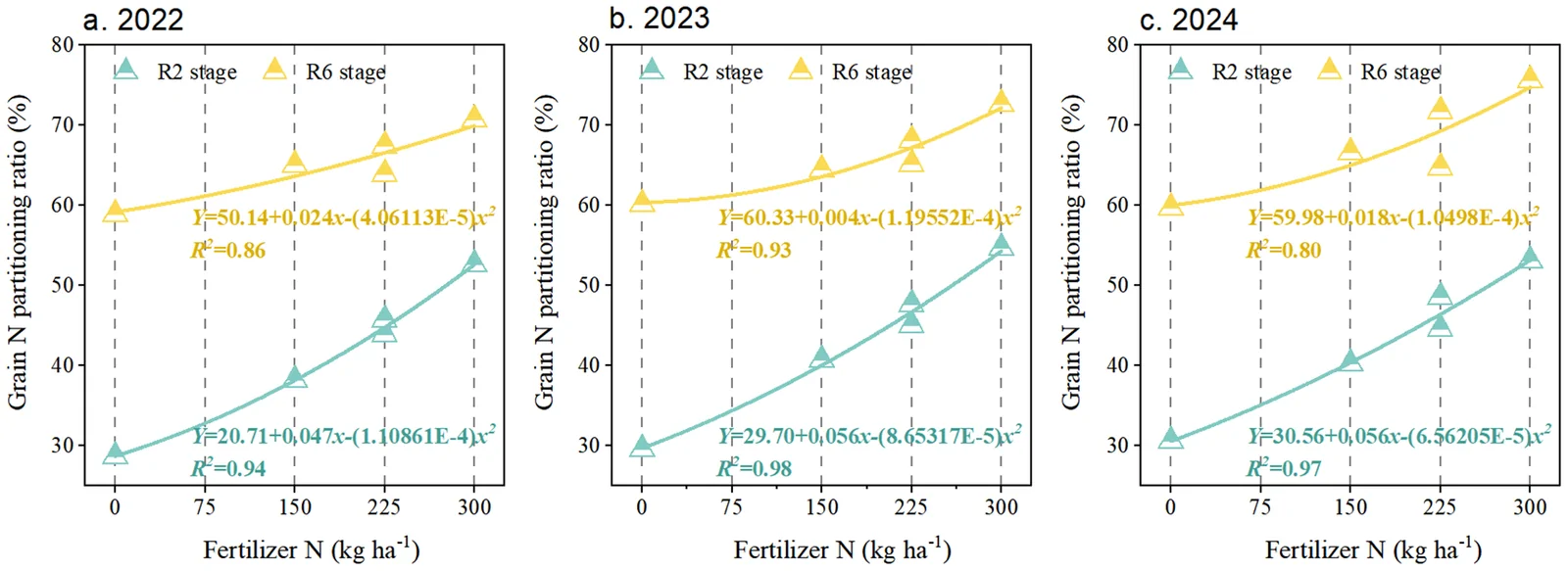
Relationship between N application rate and grain N partitioning ratio at the R2 and R6 stages in 2022 **(a)**, 2023 **(b)**, and 2024 **(c)**. Quadratic regression models were fitted to describe the relationship between N application rate and grain N partitioning ratio. NF = no N fertilizer; RF = 225kg ha^-1^ N of regular fertilizer; CF1 = 150kg ha^-1^ N of controlled-release fertilizer; CF2 = 225kg ha^-1^ N of controlled-release fertilizer; CF3 = 300kg ha^-1^ N of controlled-release fertilizer.

Regression analysis revealed a significant positive relationship between N application rate and grain N partitioning ratio at the R6 stage across the three experimental years (**Figure 5**). Grain N partitioning increased with increasing N application rate and was generally 10-15% higher at the R6 stage than at the R2 stage. The highest grain N partitioning ratio was observed under the CF3 treatment. Across the three years, CF2 and CF3 treatments maintained relatively high grain N partitioning ratios 65-75% at the R6 stage, indicating that CRFs promoted N translocation to the grains and improved the stability of N allocation among years.

### 3.3 N remobilization from vegetative tissues to grain

**Figure 6:**
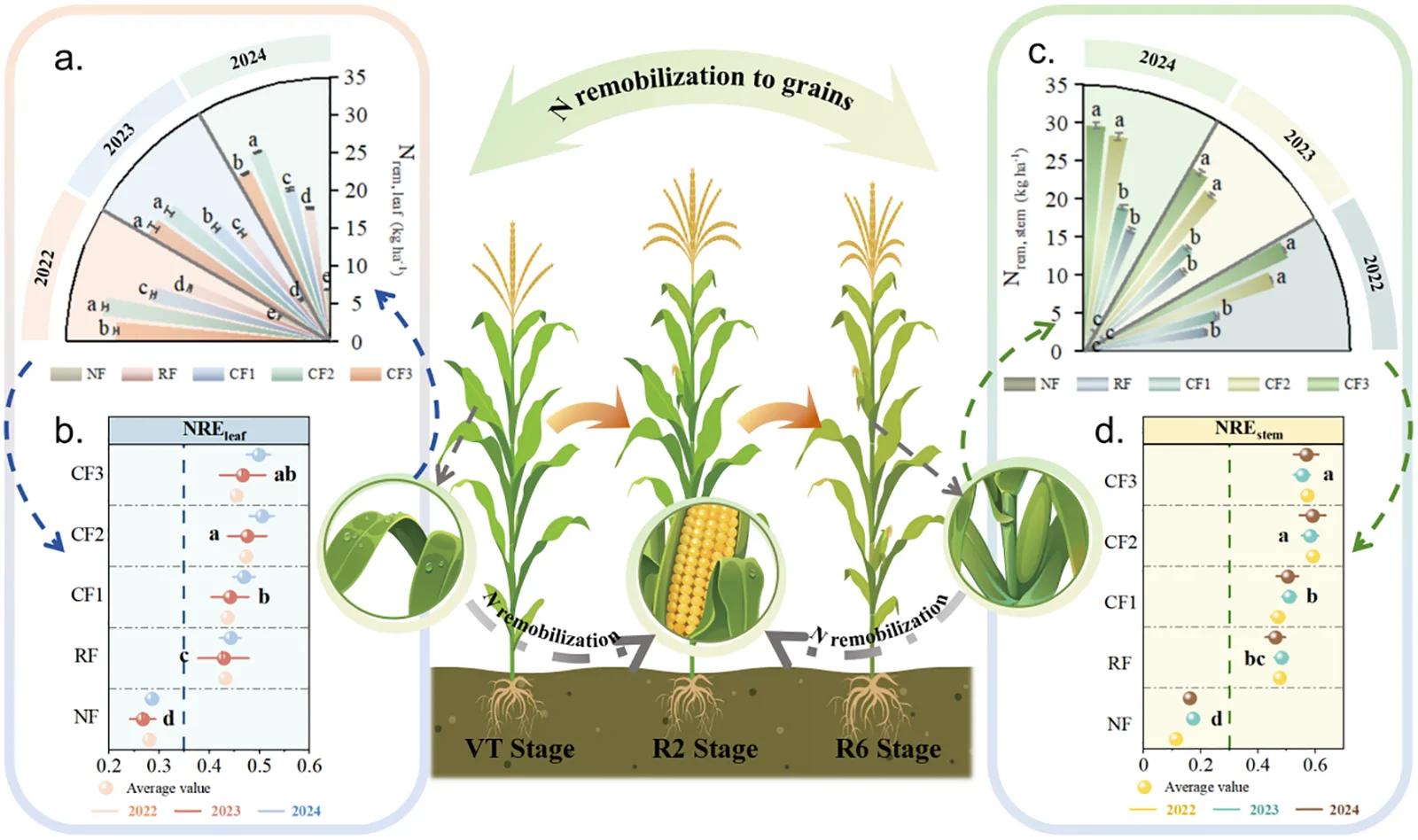
N remobilization from vegetative tissues to grains in maize under NF, RF, CF1, CF2 and CF3 treatments (2022–2024). **(a, c)** show leaf and stem N content; **(b, d)** show corresponding N remobilization efficiency (NRE). NRE_leaf_ = NRE from leaves to grains, NRE_stem_ = NRE from stems to grains. Letters indicate significant differences (p< 0.05, Tukey’s HSD). NF = no N fertilizer; RF = 225 kg ha^-1^ N of regular fertilizer; CF1 = 150 kg ha^-1^ N of controlled-release fertilizer; CF2 = 225 kg ha^-1^ N of controlled-release fertilizer; CF3 = 300 kg ha^-1^ N of controlled-release fertilizer.

N remobilization from vegetative organs to grains showed clear variation among treatments and years (**Figures 6a–d**). Overall, N remobilization varied among treatments and years, with higher N remobilization observed under CRF treatments compared with NF and RF.

In leaves, the amount of remobilized N (**Figure 6a**) was consistently higher under CRFs compared to NF and RF. Among them, CF2 and CF3 exhibited the greatest N remobilization, particularly in 2024, suggesting that improved N supply promoted the mobilization of stored N from leaves during the reproductive stage.

Similarly, NRE_stem_ (**Figure 6c**) followed a comparable pattern, with CF treatments significantly outperforming NF and RF. The magnitude of remobilized N increased markedly in 2024, highlighting the synergistic effect of optimized fertilization and favorable environmental conditions on N translocation.

The NRE further supported these patterns. NRE_leaf_ (**Figure 6b**) showed a clear ranking of CF2 ≥ CF3 > CF1 > RF > NF, with significant differences among treatments. Notably, CF2 achieved the highest NRE in most years, indicating a more efficient transfer of N from leaves to grains. NRE_stem_ (**Figure 6d**) exhibited a similar trend, with CRFs significantly improving NRE compared to NF and RF, especially in 2024. At the same N application level, the three-year average NRE_leaf_ and NRE_stem_ were 12.3 and 24.8 percentage points higher, respectively, with CF2 compared to RF. The three-year average N remobilized from leaves to grains under CRF was 3.9% units higher than from stems, whereas the three-year average NRE_stem_ was 20.7% higher than that NRE_leaf_.

### 3.4 Soil NO_3_^−^-N concentration dynamics in topsoil and subsoil

**Figure 7:**
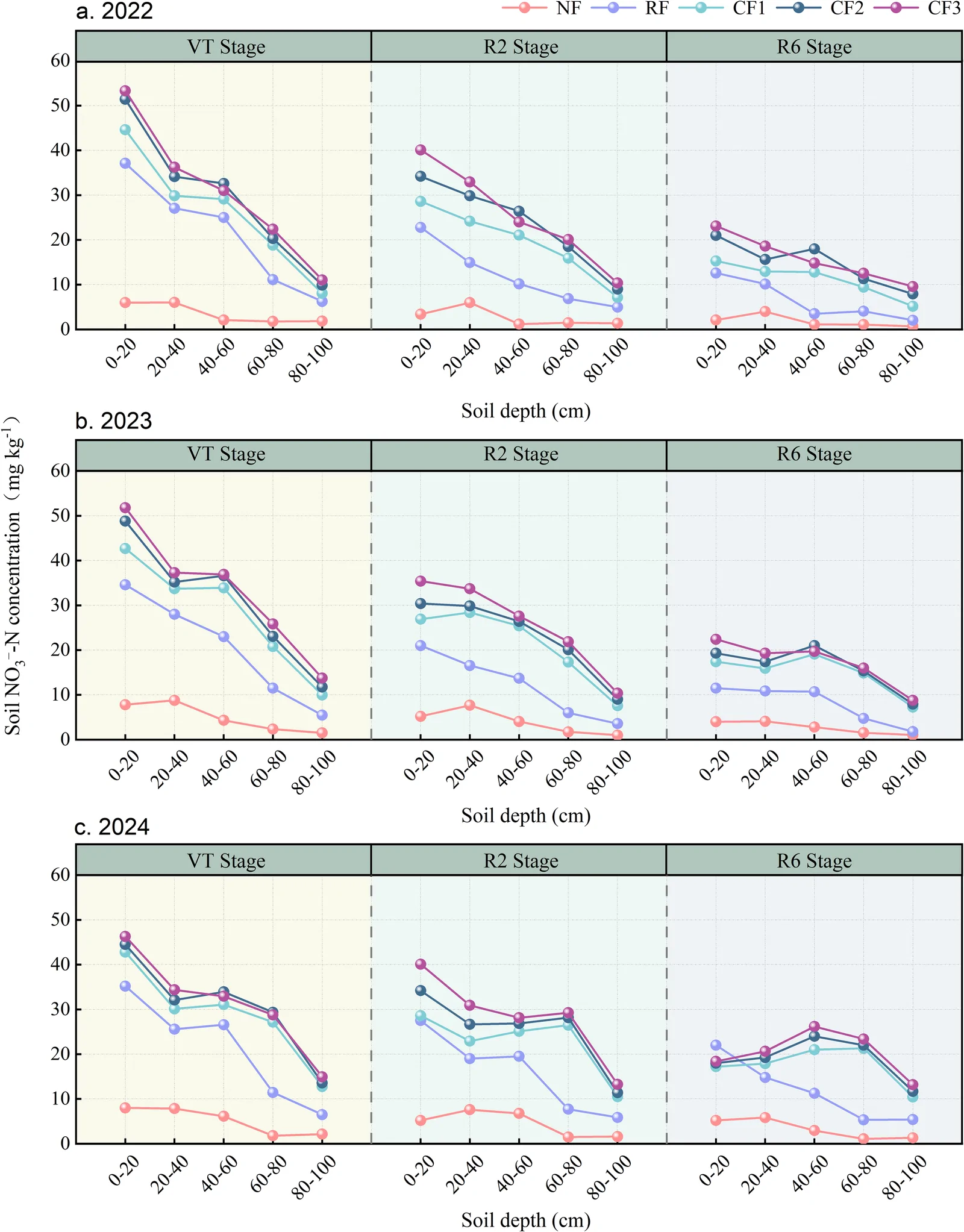
Soil NO_3_^−^-N concentration along the soil profile (0-100cm) at different growth stages (VT, R2, and R6) under different N treatments in 2022 **(a)**, 2023 **(b)** and 2024 **(c)**. NF = no N fertilizer; RF = 225 kg ha^-1^ N of regular fertilizer; CF1 = 150 kg ha^-1^ N of controlled-release fertilizer; CF2 = 225 kg ha^-1^ N of controlled-release fertilizer; CF3 = 300 kg ha^-1^ N of controlled-release fertilizer.

The NO_3_^−^-N was significantly affected by N treatment, maize growth stage and soil depth (p< 0.05). Across treatments and years, nitrate accumulation was primarily observed in the topsoil (0–40 cm), with lower concentrations generally found in deeper soil layers (40–100 cm) (**Figure 7**).

During the VT stage, soil NO_3_^−^-N concentrations were highest in the topsoil (0–20 cm) and gradually decreased with increasing soil depth under all treatments. With crop growth, nitrate levels in the surface layer declined from VT to R2, reflecting active plant N uptake. At the R2 stage, soil nitrate remained relatively higher in the upper soil layers (0–40 cm) in the fertilized treatments, particularly under CF1 and CF2.

At the R6 stage, soil NO_3_^−^-N concentrations increased in the deeper soil layers (60–100 cm), indicating downward migration of nitrate during the late growing period. This trend was more pronounced in treatments with higher N application rates. Based on the three-year average, the NO_3_^−^-N concentration in the 80–100 cm soil layer under the CF3 treatment at the R6 stage was 18.6 mg kg^-1^ higher than that under NF, while CF2 showed values 9.7 mg kg^-1^ higher than RF over the three-year average. These results suggest a greater potential risk of nitrate leaching under high-dose CRFs at later growth stages, whereas CF1 showed relatively lower NO_3_^−^-N accumulation in deep soil layers.

Interannual variation was also observed in soil nitrate distribution. Deep soil NO_3_^−^-N concentrations differed among experimental years, with lower values observed in 2022 compared with 2023 and 2024. The average NO_3_^−^-N concentration in the deep soil layer was lowest in 2022, which was 18.7% and 28.5% lower than those in 2023 and 2024, respectively.

**Figure 8:**
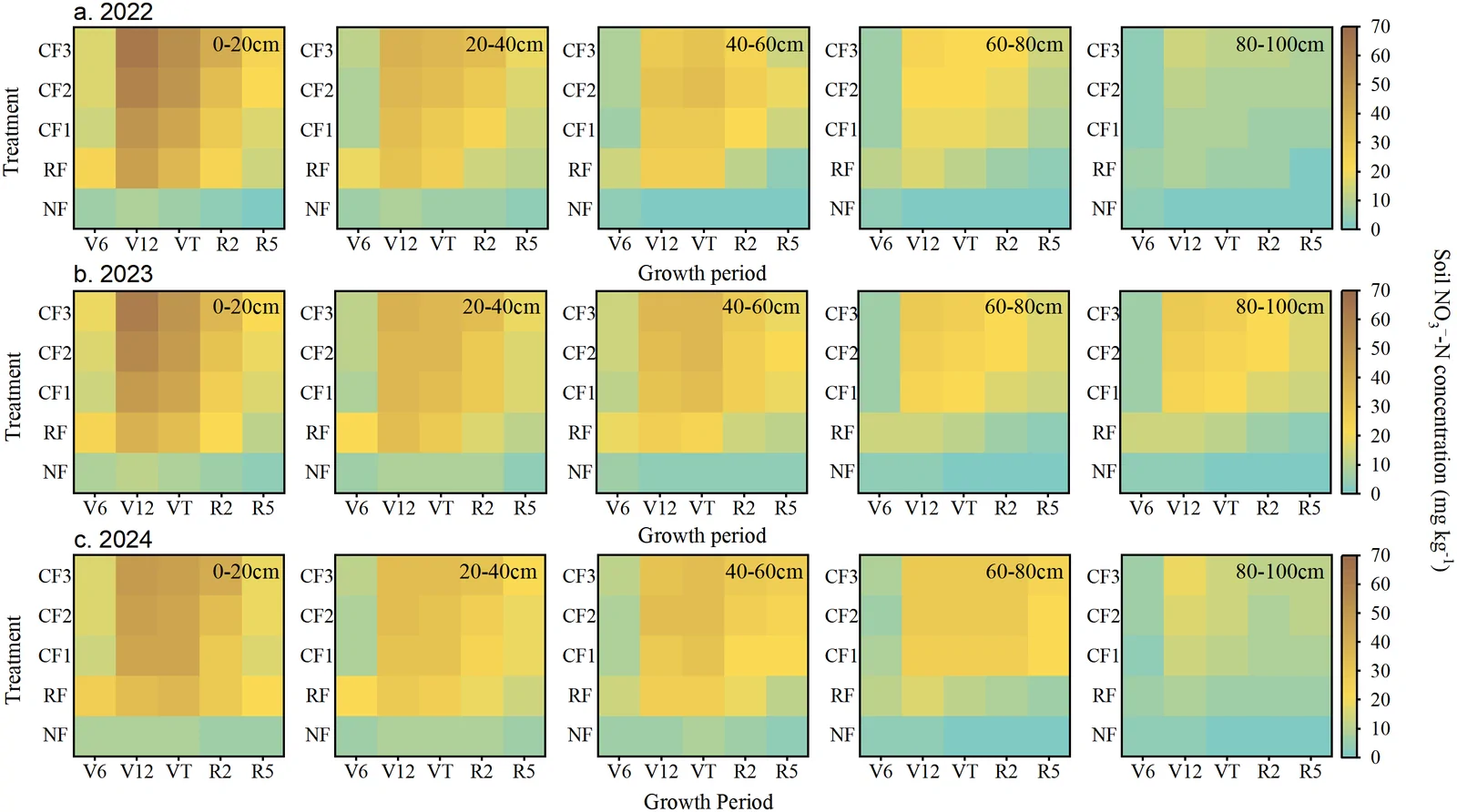
Soil NO_3_^−^-N concentration distribution during the growing season under the controlled-release fertilizer (CRF) five N application rates (NF, RF, CF1,CF2 and CF3) from 2022 **(a)**, 2023 **(b)** and 2024 **(c)**. NF = no N fertilizer; RF = 225 kg ha^-1^ N of regular fertilizer; CF1 = 150 kg ha^-1^ N of controlled-release fertilizer; CF2 = 225 kg ha^-1^ N of controlled-release fertilizer; CF3 = 300 kg ha^-1^ N of controlled-release fertilizer.

The peak NO_3_^−^-N concentration in the shallow layer (0–20 cm) could reach 65–70 mg kg^-1^ (CF3, V6), which was significantly higher than that in the deeper layer (80–100 cm) at 15 mg kg^-1^. In 2022-2024, the 20–60 cm soil layer was the critical cut-off point for the shift from NO_3_^−^-N accumulation to translocation, which began to decline significantly in the late VT, suggesting a weakening of soil re-supply in the latter part of the reproductive period (**Figure 8**). Although CFRs (especially CF3) can improve the N supply in the early stage, there is still a risk of deep accumulation if they are applied in excess (18-24% higher than RF at 80–100 cm at the R6 stage). Reasonable ratios (e.g., between CF1 and CF2, closer to CF2) can achieve a compromise between efficient utilization and lower residue.

### 3.5 Relationship between soil N and tissue N

**Figure 9:**
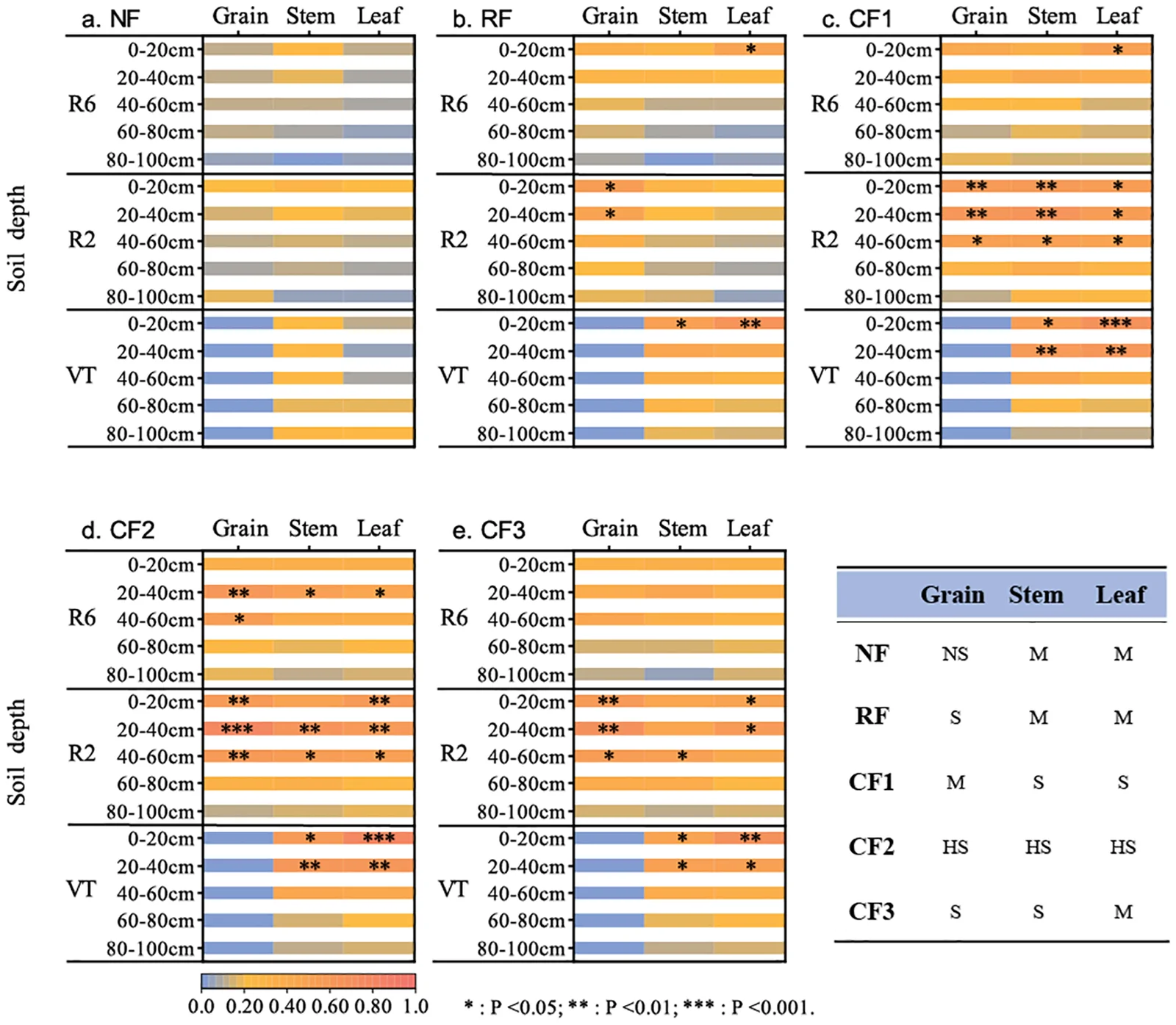
Pearson correlation coefficients between soil NO_3_^−^-N content at different soil depths (0–20, 20–40, 40–60, 60–80, and 80–100 cm) and N accumulation in maize tissues (grain, stem, and leaf) at the VT, R2, and R6 stages under different N treatments. Color scale represents the correlation coefficient (r). Asterisks denote significant correlations (*p< 0.05; **p< 0.01; ***p< 0.001). The table summarizes the overall significance level of correlations for each treatment (NS = non-significant; M = moderate; S = significant; HS = highly significant). **(a)** NF = no N fertilizer; **(b)** RF = 225 kg ha^-1^N of regular fertilizer; **(c)** CF1 = 150 kg ha^-1^ N of controlled-release fertilizer; **(d)** CF2 = 225 kg ha^-1^ N of controlled-release fertilizer; **(e)** CF3 = 300 kg ha^-1^ N of controlled-release fertilizer.

Grain N accumulation during the reproductive stage was more closely related to soil NO_3_^−^-N availability measured at silking than at maturity. N partitioning to grain was primarily coupled with N availability in the 20–60 cm soil layer under CRF (**Figure 9**). The coupling between subsoil NO_3_^−^-N and grain N accumulation was stronger under controlled-release fertilization than under urea application.

### 3.6 Soil–plant N supply–demand coupling

**Figure 10:**
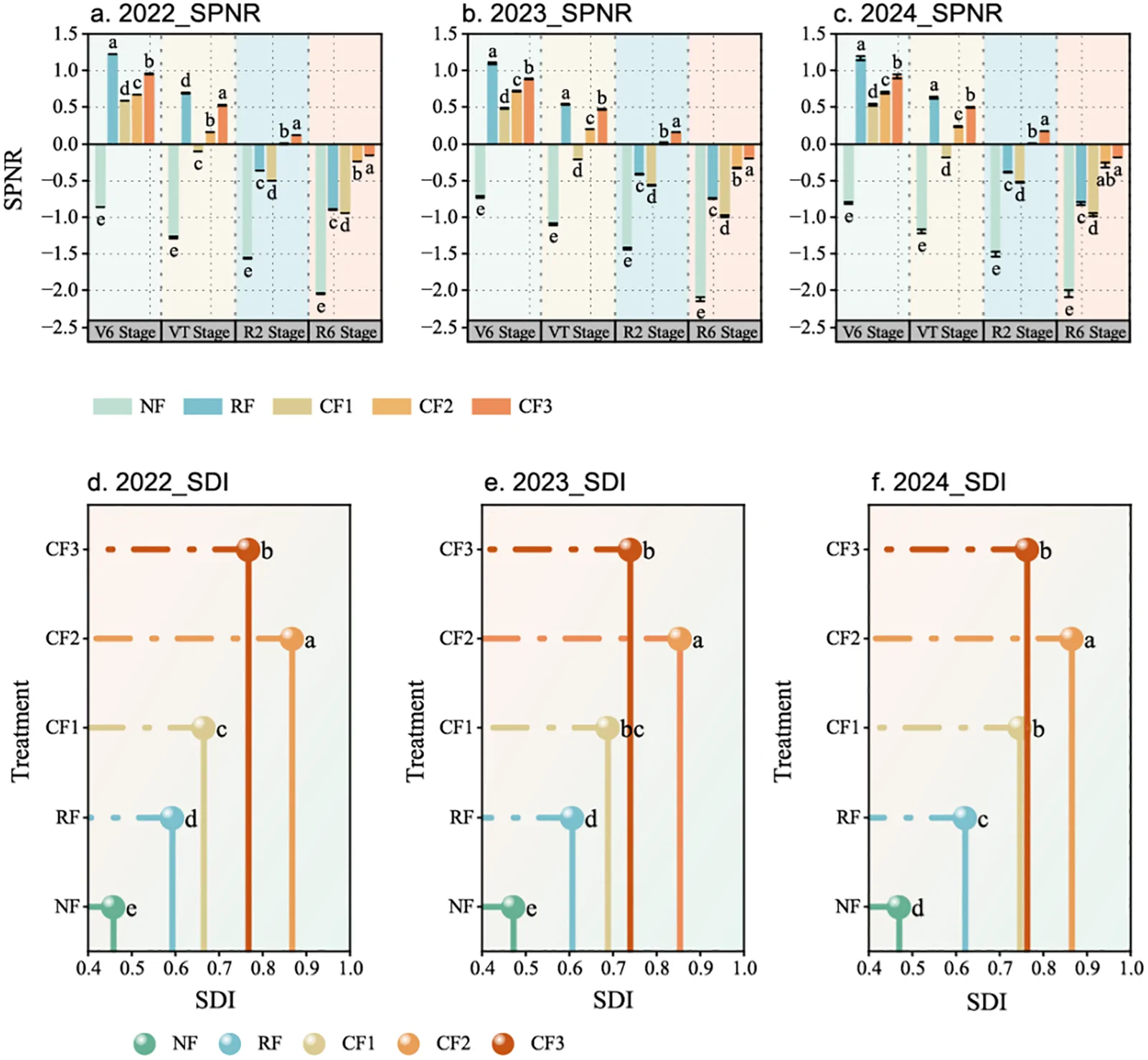
Seasonal dynamics of supply–plant N requirement synchronization (SPNR) and supply-demand synchrony index (SDI) under different fertilizer treatments from 2022 to 2024. **(a–c)** show SPNR at V6, VT, R2, and R6 growth stages in 2022 **(a)**, 2023 **(b)**, and 2024 **(c)**. **(d–f)** show SDI in 2022 **(d)**, 2023 **(e)**, and 2024 **(f)**. Treatments are NF, RF and CF1-CF3. Letters indicate significant differences among fertilizer treatments at p< 0.05. NF = no N fertilizer; RF = 225 kg ha^-1^ N of regular fertilizer; CF1 = 150 kg ha^-1^ N of controlled-release fertilizer; CF2 = 225 kg ha^-1^ N of controlled-release fertilizer; CF3 = 300 kg ha^-1^ N of controlled-release fertilizer.

Across the three years (**Figure 10**), CRFs (CF2 and CF3) markedly improved the synchronization between maize N demand and supply compared with RF and NF. The three-year average SPNR at the V6 stage was highest under CF3 and CF2, significantly exceeding RF and NF, indicating better early-stage N availability. At reproductive stages (R2 and R6), CF2 maintained moderate positive SPNR values, whereas NF showed strongly negative SPNR (down to -1.8), reflecting insufficient N supply during critical growth periods.

Similarly, the average SDI values for CF3 and CF2 were 0.77 and 0.86, respectively, compared with 0.61 for RF, indicating that CRF application increased overall N supply–demand synchronization by 26.2% to 41.0% relative to RF. Interannual variation revealed that 2023, with moderate rainfall, exhibited the highest SPNR and SDI across treatments. In 2024, CF3 showed lower synchronization indices compared with 2023. Soil–plant N ratios were high at early vegetative stages, indicating excess soil N relative to crop demand, but decreased at VT and R2, suggesting improved synchronization.

These results indicate that CF2 can optimize N supply throughout the maize growth cycle, achieving high SPNR and SDI while minimizing excessive N release and potential environmental risk-a strategy that outperforms RF in both efficiency and sustainability.

### 3.7 N synchronization drives yield formation

**Figure 11:**
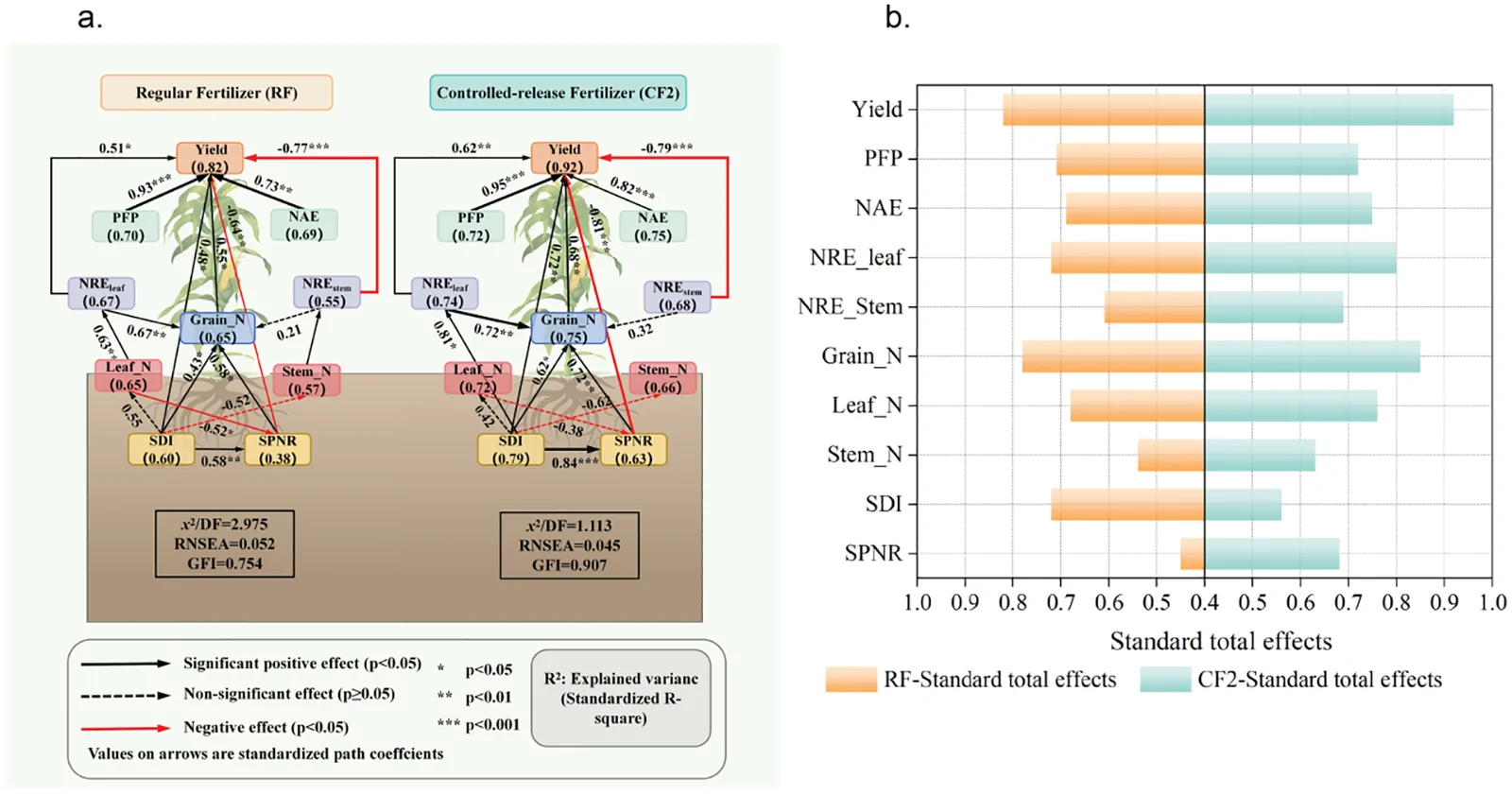
Structural equation model illustrating the relationships among N supply intensity (SDI), supply–demand synchronization (SPNR), plant N allocation, remobilization (NRE), and yield formation. Solid lines indicate significant effect and dotted lines indicate non-significant effect. Negative and positive effects are indicated by blue and red arrows, respectively, and the numbers next to the arrows indicate the path coefficients, with the width of the arrows positively correlated with the path coefficients. *, **, and *** indicate significant effect at 0.05, 0.01, and 0.001 level, respectively. **(a)** Structural equation models under RF (Regular Fertilizer) and CF2 (Controlled-release Fertilizer) treatments; **(b)** Comparison of standard total effects between RF and CF2 treatments.

Path coefficient structure models (SEMs) were constructed using data from ten variables, which included SDI, SPNR, plant N allocation, and NRE under different CRF fertilizers from 2022 to 2024 (**Figure 11a**). The model explained a higher proportion of yield variance under controlled-release fertilization (CF2; R^2^ = 0.79) than regular fertilization (RF; R^2^ = 0.74). Under RF, SDI significantly affected SPNR, but its downstream effects on NRE and internal N cycling were relatively weak. In contrast, CF2 strengthened the linkages among SPNR, N allocation, and N remobilization, leading to more efficient N transfer to grain. Standardized total effects showed that yield had the highest contribution (>0.8), followed by grain N and NRE-related traits, with consistently greater effects under CF2 than RF (**Figure 11b**), The SEM analysis showed that SPNR had stronger associations with grain N accumulation and yield formation under CF2 than under RF (R^2^ = 0.92, GIF = 0.907).

Under RF treatment, yield formation was mainly driven by N SDI through vegetative N accumulation and subsequent remobilization. In contrast, under CF2 treatment, SPNR became the dominant factor, directly enhancing grain NA and yield formation.

## 4 Discussion

### 4.1 CRFs vs. RF yield enhancement through optimized N synchronization

This study demonstrated that the application of CRFs, particularly at the CF2 rate, significantly enhanced maize grain yield and NUE compared to conventional urea. These improvements are consistent with previous findings that CRFs can synchronize N release with crop demand, thereby reducing nutrient losses and improving uptake efficiency (Lee and Ku, 2025; Li et al., 2020). The enhanced performance under CRF treatments was primarily attributed to the alignment between N release kinetics and maize growth stages, especially during reproductive development, which facilitated sustained N uptake and promoted remobilization from vegetative tissues to grains (Guo et al., 2022; Ciampitti et al., 2013). Soil NO_3_^−^-N dynamics revealed that while CRFs improved temporal N supply, excessive N input (CF3) led to NO_3_^−^-N accumulation in deeper soil layers (60–100 cm), indicating a heightened risk of leaching. This observation aligns with studies showing that polymer-coated urea can delay N release, but under high application rates, residual N may exceed root uptake capacity, leading to environmental losses (Bundy and Andraski, 2007);. These findings underscore the importance of optimizing CRF application rates to balance productivity with environmental sustainability in irrigated arid cropping systems (Azeem, 2026; Ji et al., 2025; Zhang et al., 2026).

The improved NAE and PFP observed under CF1 and CF2 treatments can be attributed to the gradual release characteristics of polymer-coated urea, which better matched the temporal N requirements of maize, particularly from the VT to R2 stages (Kalita and Vaid, 2026). During this period, maize exhibits rapid biomass accumulation and high N demand to support reproductive development. Sustained N availability under CRF facilitated continuous assimilation, resulting in greater total N uptake and higher biomass, consistent with the work (Li et al., 2021; Xiao et al., 2024; Zhang et al., 2026). In contrast, rapid-release fertilizers (RF) provided an early N pulse but failed to maintain adequate supply later in the season, leading to physiological N stress, as previously reported by Geng et al. (2016) and Ji et al. (2021). Furthermore, the enhanced NAE and PFP under CF1 and CF2 relative to RF indicate that moderate CRF use enabled maize to produce more grain per unit of applied N. This supports the conceptual framework that optimized N supply reduces physiological N dilution and enhances photosynthetic NUE, thereby improving the conversion of absorbed N into productive biomass (Mu et al., 2016). However, future studies should integrate economic analyses with agronomic and environmental assessments to evaluate the overall feasibility of large-scale adoption of CF2 treatment. Beyond conventional NUE indicators, the synchronization indices developed in this study provide a process-based perspective for evaluating fertilizer performance. While NUE metrics quantify the efficiency of N conversion into crop biomass or grain, SDI and SPNR reveal whether N supply is temporally aligned with crop demand. This distinction is particularly important for controlled-release fertilizers, where improving synchronization rather than simply increasing N availability determines sustainable N management outcomes.

### 4.2 Tissue-level N partitioning, remobilization, and internal cycling: CRF advantages over RF

A major contribution of this study was the detailed characterization of tissue-level N partitioning and N remobilization dynamics under CRF management. Compared with RF, CRFs substantially increased N redistribution from vegetative tissues to grains during reproductive development (Chen et al., 2016; Wang et al., 2025). At the same N application rate, CF2 increased N transfer from leaves and stems to grains by 15.3%, while grain N accumulation increased by approximately 20%, demonstrating that CRFs significantly enhanced NRE and grain filling capacity (Cao et al., 2021). Tissue-level N partitioning analysis revealed that CRFs played a crucial role in enhancing N redistribution to reproductive tissues during grain filling (Ren et al., 2025b). From VT to R6, CRF treatments showed a greater increase in grain N proportion and a sharper decline in leaf and stem N compared with RF, indicating more effective remobilization of stored N (Zhang et al., 2025). This is consistent with findings by Ciampitti et al. (2013) and Martinez et al. (2021), who reported that CRFs improve internal N dynamics and remobilization efficiency during reproductive stages.

N remobilization is a critical determinant of grain filling and yield formation in maize (Ciampitti and Vyn, 2013). During reproductive development, maize progressively reallocates N from vegetative tissues to reproductive tissues (Fan et al., 2023). N remobilization during reproductive growth represents a key physiological process determining maize grain filling, particularly when external N supply becomes insufficient to fully support grain N demand (Masclaux-Daubresse et al., 2008; Ciampitti and Vyn, 2013). Previous studies have demonstrated that approximately 40-60% of grain N in maize may originate from remobilization of N accumulated before anthesis, with leaves and stems acting as major N reservoirs during the grain-filling period (Masclaux-Daubresse et al., 2010). In this study, the enhanced NRE_leaf_ and NRE_stem_ under CRF treatments suggest that improved temporal synchronization of N availability not only increased N uptake but also delayed premature N depletion from vegetative tissues, thereby maintaining a stronger N source capacity during grain filling. This finding is consistent with previous reports that prolonged photosynthetic activity and delayed leaf senescence contribute to improved N translocation efficiency and higher grain N accumulation in maize (Chen et al., 2016; Hirel et al., 2007).

In the present study, CRFs accelerated this redistribution process, resulting in higher NRE in both leaves and stems. In the present study, controlled-release fertilization enhanced N redistribution from leaves and stems to grains, indicating that improved N synchronization strengthened internal N cycling during reproductive growth (Ma et al., 2025). Compared with RF, CRFs maintained greater remobilization efficiency in both leaves and stems, indicating that synchronized N supply promoted more effective utilization of vegetative N reserves during grain filling (Qiao et al., 2025). The results also suggest functional differences between leaves and stems as N sources during reproductive development. Although leaves directly supported grain filling through active remobilization, stems appeared to function as important temporary N storage pools under synchronized fertilization conditions (Ren et al., 2025b). Kong et al. (2016) similarly reported that stem tissues serve as major reservoirs of remobilizable N when grain N demand intensifies during reproductive growth. This buffering role partly explain the stronger NRE observed under CRF treatments.

Notably, CF2 consistently maintained high grain N partitioning (65–75%) across all years, suggesting that medium CRF application effectively supported both sustained uptake and efficient internal redistribution. In contrast, CF3 also increased grain N proportion but was accompanied by excess residual soil N, indicating that enhanced partitioning under high N does not necessarily imply efficient system-level N use (Zhang et al., 2023a; Zhao et al., 2026). These results added mechanistic clarity on how CRFs influence organ-specific N allocation patterns.

### 4.3 Soil N dynamics and environmental implications

Soil NO_3_^−^-N profiles demonstrated clear stratification, with higher concentrations in the 0–40 cm layer during early vegetative stages and downward migration toward 60–100 cm by R6. This pattern indicates that N release from CRF may exceed crop uptake capacity during the late growth period, especially under high N input (CF3). Consequently, CF3 exhibited significantly nitrate accumulation in deep soil layers than CF2 and RF, indicating an increased potential risk of nitrate movement beyond the active root zone. These findings are consistent with previous studies showing that excessive N application, even with CRFs, may promote nitrate residual accumulation and leaching under irrigated conditions (Janke et al., 2022; Nasini et al., 2013; Wilson et al., 2010). Recent research has further emphasized that the environmental benefits of CRFs largely depend on the synchronization between fertilizer N release and crop N demand. When N release is well matched with plant uptake, CRFs can improve N availability and reduce potential losses. However, excessive N inputs may disrupt this synchronization by causing N release to exceed crop demand, leading to residual nitrate accumulation even under controlled-release conditions. Therefore, the elevated NO_3_^−^-N concentration at 60–100 cm under CF3 in the present study indicates that excessive CRF application may compromise the synchronization advantage and increase the potential risk of nitrate migration beyond the active root zone.

However, soil-plant N synchronization is also influenced by environmental conditions, including precipitation, temperature, and irrigation management (Kirnak et al., 2013). Variations in precipitation and temperature affect soil N transformation, nitrate transport, and crop N demand, while irrigation scheduling regulates soil moisture and nitrate availability in the root zone (Bijay-Singh and Craswell, 2021). Therefore, interannual differences in SDI and SPNR may partly reflect climatic variability in addition to fertilizer treatments. Previous studies have demonstrated that interactions between water availability and N management strongly regulate maize N uptake efficiency and soil NO_3_^−^-N retention (Guo et al., 2022). Recent advances in precision fertilization have highlighted that fertilizer management strategies should be evaluated together with irrigation conditions because soil moisture regulates both N diffusion and nitrate transport processes. Akhtar et al. (2025) further demonstrated that improved fertilizer placement and controlled N release could enhance crop N acquisition while mitigating N losses, highlighting the importance of coordinating fertilizer release patterns with soil water dynamics. Therefore, the interannual variation observed in SDI and SPNR in the present study may reflect not only differences among fertilizer treatments but also the interaction between climatic conditions and N management strategies.

The identification of the 20–60 cm layer as a critical zone for N retention reinforces the importance of aligning fertilizer release characteristics with root-zone nutrient absorption dynamics. Moderate CRF inputs (CF1-CF2) maintained sufficient N in the active root layer while minimizing late-season surplus, consistent with sustainable N management principles outlined by Qiang et al. (2020) and Xue et al. (2024). The use of ^15^N isotope tracing in future experiments would enable clearer separation of absorbed versus remobilized N pools, as demonstrated by Kalu et al. (2021), and Fenilli et al. (2007). Additionally, evaluating CRF release behavior under contrasting irrigation regimes and climatic scenarios will be necessary to optimize CRF×drip irrigation synergies and improve the robustness of synchronization-based N management strategies (Ji et al., 2025). Future studies should also consider integrating CRFs with smart fertilization systems that dynamically adjust N supply according to real-time plant nutritional status and soil N monitoring, as proposed by Liu et al. (2025) and Sankati et al. (2024).

Although this work provides robust evidence on CRF impacts via N uptake, partitioning, soil distribution, and SEM pathways, limitations remain. It did not directly quantify N translocation using isotopes, further experiments with ^15^N tracing would better separate absorbed versus remobilized pools (Kalcsits et al., 2014; Braun et al., 2018). In addition, because conventional and CRFs differed in formulation and release characteristics, the present design cannot completely separate the effects of fertilizer type from N rate. However, the comparison between RF and CF2 under the same N input (225 kg N ha^-1^) suggested that the improved N synchronization was primarily associated with controlled-release characteristics rather than N quantity alone. The potential environmental impacts of polymer coatings should also be considered. Although polymer-coated fertilizers can improve NUE by regulating nutrient release and reducing N losses, some non-biodegradable coating materials may persist in agricultural soils and contribute to microplastic accumulation. Therefore, future studies should integrate long-term assessments of coating degradation, polymer residue dynamics, and N cycling processes, together with factorial experiments using identical N rates and contrasting release patterns, to comprehensively evaluate the agronomic and environmental sustainability of polymer-coated fertilizers. Moreover, multi-environment trials incorporating varied irrigation regimes and continuous monitoring of soil and plant N status are needed to improve the applicability of synchronization-based N management strategies (Shahab et al., 2025; Suwardi et al., 2023).

## 5 Conclusion

This study investigated the effects of CRFs on maize yield, NAE, PFP, and soil-plant N supply-demand relationships over three years. Results showed that CRFs, particularly at moderate rates (CF2, 225kg ha^-1^), optimize maize yield and N use by enhancing the synchronization between soil N supply and plant demand. CF2 increased grain N accumulation through improved leaf and stem N remobilization, while achieving higher grain N partitioning compared with RF, indicating more efficient internal N cycling. However, future studies should evaluate its economic feasibility as relevant consideration for the practical adoption of CRFs at large-scale. Excessive application (CF3) enhanced early N supply but caused deeper soil nitrate accumulation, highlighting potential leaching risks. SEM revealed that CRFs shift yield regulation from N supply intensity to synchronization-driven control, aligning soil N availability with crop demand (SDI 0.77–0.86 vs. 0.61 for RF). Overall, the integration of whole-season N balance with tissue-scale N partitioning reveals that the benefits of CRFs extend beyond yield enhancement to include more efficient internal N utilization and reduced environmental risk—provided that application rates are carefully optimized. These findings indicate that moderate CRF use, rather than high N inputs, represents a sustainable strategy for maize production in arid and semi-arid irrigated systems.

## Data Availability

The original contributions presented in the study are included in the article/supplementary material. Further inquiries can be directed to the corresponding author.
